# The Expression of RAAS Key Receptors, *Agtr2* and *Bdkrb1*, Is Downregulated at an Early Stage in a Rat Model of Wolfram Syndrome

**DOI:** 10.3390/genes12111717

**Published:** 2021-10-28

**Authors:** Marite Punapart, Kadri Seppa, Toomas Jagomäe, Mailis Liiv, Riin Reimets, Silvia Kirillov, Allen Kaasik, Lieve Moons, Lies De Groef, Anton Terasmaa, Eero Vasar, Mario Plaas

**Affiliations:** 1 Laboratory Animal Centre, Institute of Biomedicine and Translational Medicine, University of Tartu, 14B Ravila Street, 50411 Tartu, Estonia; kadri.seppa@ut.ee (K.S.); toomas.jagomae@gmail.com (T.J.); riin.reimets@ut.ee (R.R.); silvia.kirillov@ut.ee (S.K.); anton.terasmaa@ut.ee (A.T.); 2 Department of Physiology, Institute of Biomedicine and Translational Medicine, University of Tartu, 19 Ravila Street, 50411 Tartu, Estonia; eero.vasar@ut.ee; 3 Department of Pharmacology, Institute of Biomedicine and Translational Medicine, University of Tartu, 19 Ravila Street, 50411 Tartu, Estonia; mailis.liiv@ut.ee (M.L.); allen.kaasik@ut.ee (A.K.); 4 Research Group Neural Circuit Development and Regeneration, Department of Biology, University of Leuven, Naamsestraat 61, Box 2464, 3000 Leuven, Belgium; lieve.moons@kuleuven.be (L.M.); lies.degroef@kuleuven.be (L.D.G.)

**Keywords:** Wolfram syndrome, *Wfs1*, *Wfs1* knock-out, liraglutide, valproic acid, RAAS, aldosterone, bradykinin, *Agtr2*, *Bdkrb1*

## Abstract

Wolfram syndrome (WS) 1 is a rare monogenic neurodegenerative disorder caused by mutations in the gene encoding WFS1. Knowledge of the pathophysiology of WS is incomplete and to date, there is no treatment available. Here, we describe early deviations in the renin-angiotensin-aldosterone system (RAAS) and bradykinin pathway (kallikrein kinin system, KKS) observed in a rat model of WS (*Wfs1* KO) and the modulative effect of glucagon-like peptide-1 receptor agonist liraglutide (LIR) and anti-epileptic drug valproate (VPA), which have been proven effective in delaying WS progression in WS animal models. We found that the expression of key receptors of the RAAS and KKS, *Agtr2* and *Bdkrb1*, were drastically downregulated both in vitro and in vivo at an early stage in a rat model of WS. Moreover, in *Wfs1*, KO serum aldosterone levels were substantially decreased and bradykinin levels increased compared to WT animals. Neither treatment nor their combination affected the gene expression levels seen in the *Wfs1* KO animals. However, all the treatments elevated serum aldosterone and decreased bradykinin in the *Wfs1* KO rats, as well as increasing angiotensin II levels independent of genotype. Altogether, our results indicate that *Wfs1* deficiency might disturb the normal functioning of RAAS and KKS and that LIR and VPA have the ability to modulate these systems.

## 1. Introduction

Wolfram syndrome (WS) is a rare monogenic progressive neurodegenerative disease mainly characterized by juvenile-onset diabetes mellitus, diabetes insipidus, loss of vision due to optic nerve atrophy, sensorineural deafness and retinal ganglion cell death [1,2]. All of these symptoms are also characteristic of the rat model of WS (*Wfs1* KO) described by our research group [3].

To date, no treatment is available for WS, although there are some promising drugs against the progression of WS—two of which are the glucagon-like peptide-1 receptor (GLP-1R) agonist liraglutide (LIR), used for the treatment of type 2 diabetes [4,5], and mood stabilizer valproic acid (VPA). It has been shown that the GLP-1R agonists LIR and exenatide delay diabetic phenotype in WS rats and mice [6,7,8] and that dulaglutide improves glycemic control of WS patients [9]. Furthermore, we have found that long-term treatment with LIR, neurotrophic factor 7,8-dihydroxyflavone or their combination has a neuroprotective effect, prevents cognitive decline through multiple processes, improves learning, and delays the loss of visual acuity in aged WS rats [10,11]. VPA is known to induce WFS1 expression and to modulate ER stress response, possibly through the activation of WFS1 [12,13,14]. Moreover, in vitro VPA reduces apoptosis in cell lines carrying autosomal dominant WFS1 mutations and acute treatment with VPA improves glucose tolerance in *Wfs1*-deficient mice [15,16]. Nevertheless, it is not fully known how LIR and VPA inhibit WS progression.

Both LIR and VPA have been shown to affect the expression of components of the renin-angiotensin-aldosterone system (RAAS) [17,18], which controls important physiological functions, including sodium and water balance and the regulation of blood pressure. The deregulation of RAAS is implicated in the pathophysiology of many diseases, including pulmonary fibrosis, cancer, diabetes and neurodegeneration [19,20,21,22]. Importantly, in addition to well-known systemic RAAS, there is a local tissue-specific RAAS, including in the brain and pancreas, controlling vasodilation, vasoconstriction, proliferation, regeneration, cellular stress and inflammatory response [23,24,25,26].

RAAS performs its complex functions through a cascade of peptides. The precursor angiotensinogen is converted into angiotensin I (Ang I) by the aspartyl-protease renin, which is released from the renal juxta-glomerular cells in response to a drop in blood pressure [27]. Ang I is further processed by angiotensin-converting enzyme (ACE) into angiotensin II (Ang II), the central peptide hormone of RAAS, which acts through AGTR1 (Ang II type 1) and AGTR2 (Ang II type 2) receptors. AGTR1 activation by increased Ang II production contributes to elevated blood pressure, water retention, aldosterone release, proliferation, and pro-inflammatory and pro-fibrotic responses. AGTR2 receptor activation displays the opposite effect, resulting in vasodilation [28]. Ang I can also be processed by angiotensin-converting enzyme 2 (ACE2) to heptapeptide Ang-(1–9), which in turn is converted to Ang-(1–7) by ACE. Ang-(1–7) is the endogenous ligand for vasodilating G protein-coupled receptor MAS1 proto-oncogene, which, similarly to AGTR2, acts as a functional antagonist of AGTR1 [19,29,30]. Besides vasodilation, the activation of MAS1 and AGTR2 leads to nitric oxide release, subsequent hyperpolarization via the activation of potassium channels, anti-inflammatory and anti-fibrotic responses, the inhibition of proliferation and, eventually, to apoptosis [19]. Moreover, AGTR2 expression tonically inhibits ACE activity [31] and promotes axonal regeneration in the optic nerve of adult rats [32].

The bradykinin (BK) of the kallikrein-kinin system (KKS) is another regulator of blood pressure and inflammation and is tightly intertwined with RAAS through kallikrein and ACE [33]. There are two subtypes of BK receptors, bradykinin receptor 1 and 2 (BDKRB1 and BDKRB2, respectively). Similarly to AGTR2 stimulation, BK-induced activation of BDKRB2 results in vasodilation, hypotension and natriuresis. BDKRB1 activation is induced by injury, pain and strong inflammatory responses [34,35]. In the CNS, BDKRB1 mediates Ca^2+^–dependent BK-induced microglial migration, which depends on Ca^2+^ influx via reverse mode of Na^+^/Ca^2+^ exchanger NCX1 [36]. BK receptor signaling is enhanced by both Ang-(1–9) and Ang-(1–7) through the modification of ACE activity and BDKRB2 sensitivity [37]. BK is degraded by ACE and if ACE activity is reduced, the BK-directed hypotensive axis is more active [38,39].

The WFS1 protein is widely expressed in various tissues, with higher levels in the brain, pancreas, lung, heart and retina [40,41,42,43], which is why WS is a complex disease affecting many organ systems. WFS1 protein is associated with the regulation of endoplasmic reticulum stress response and calcium homeostasis [44,45], but there is limited knowledge of its exact function and interactions. The role of the RAAS and bradykinin pathways suggests that they may be implicated in the development of WS, but they have never been studied in WS animal models.

The two main organs involved in systemic RAAS are the lungs and heart, which also feature high WFS1 expression. This abundant expression indicates that WFS1 must perform a relevant function in those tissues. Moreover, in addition to the main pathophysiology, heart and lung problems have been described in WS patients. There are reports that a significant number of WS patients experience congenital heart disease, such as sinus tachycardia, atrial or ventricular arrhythmias, ventricular septal defects or pulmonary valvular stenosis [46,47]. WS patients often suffer from respiratory issues and breathing problems, mostly of neurological origin, but cases cases of early-onset respiratory problems before the development of vision issues, type 1 diabetes and neurogenic bladder have also been described [48].

Thus, the aim of the current study was to investigate possible deviations in the RAAS and bradykinin pathways in the heart and lungs of a rat model of WS at an early stage, before the development of the main symptoms. Furthermore, as LIR and VPA have shown promising results against WS progression and the ability to affect RAAS components, the rats were treated with LIR, VPA or their combination for eight consecutive days to evaluate whether those compounds act by modulating the RAAS and bradykinin pathways in *Wfs1* deficiency.

## 2. Materials and Methods

### 2.1. Animals

The generation and phenotype of a WFS1 mutant (*Wfs1* exon 5 knock-out) rat has been described previously [3]. For this study, 3.5–4-month-old outbred CD® (Sprague-Dawley) IGS male homozygous *Wfs1*-deficient (*Wfs1*-ex5-KO232) and wild-type littermate (as a control) rats were used. The outbred rat line was chosen for better translational value. The breeding and genotyping were performed at the Laboratory Animal Centre at the University of Tartu. The animals were housed in cages, in groups of four animals per cage, under a 12 h light/dark cycle (lights on at 7 a.m.). The rats enjoyed unlimited access to food and water. Sniff universal mouse and a rat maintenance diet (Ssniff #V1534, ssniff Spezialdiäten, Germany) and reverse osmosis-purified water were used. The experiments were performed between 9 a.m. and 5 p.m. All the experimental protocols were approved by the Estonian Project Authorization Committee for Animal Experiments (No 165, 3 April 2020), and all the experiments were performed in accordance with the European Communities Directive of September 2010 (2010/63/EU). The study was carried out in compliance with the ARRIVE guidelines.

### 2.2. Repeated Liraglutide and Valproate Treatment

The rats were randomly allocated liraglutide (LIR, *n* = 8), valproate (VPA, *n* = 8), liraglutide + valproate co-treatment (LIR + VPA, *n* = 8), or saline (vehicle) for the control group (SAL, *n* = 8). The animals received a daily dose of LIR (0.4 mg/kg/day; Novo Nordisk, Denmark), VPA (0.3 g/kg/day; valproic acid sodium salt, Sigma-Aldrich P4543), LIR + VPA, or SAL (0.9% NaCl, saline) for eight consecutive days. All the drugs were dissolved in saline and injected subcutaneously at a volume of 1 mL/kg. Effective and safe doses of the drugs were chosen based on our previous studies [6].

### 2.3. Sample Collection

After eight days of treatment and within 4 h of the last injection, the animals were killed with an intraperitoneal injection of Euthasol vet (300 mg/kg). The lower lobe of the lung and left ventricle of the heart were dissected, immediately washed with saline and snap frozen in liquid nitrogen. The trunk blood from each animal was collected and the serum separated as described previously [6]. The tissue samples and blood serum were stored at −80 °C for further analysis of relative gene expression and peptide levels, respectively.

### 2.4. Cell Culture of Rat Primary Cortical Neurons

Primary cultures of rat cortical neurons for the *Wfs1* siRNA experiments were prepared from the brains of neonatal Wistar rats (Wistar Hannover, Taconic, Rensselaer, NY, USA) less than a day old, as described earlier [49]. Briefly, cortices were dissected in ice-cold Krebs–Ringer solution containing 0.3% bovine serum albumin and then trypsinized in 0.8% trypsin for 10 min at 37 °C, following trituration in a 0.008% DNase solution containing 0.05% soybean trypsin inhibitor. The cells were resuspended in Basal Medium Eagle with Earle’s salts (BME) containing 10% heat-inactivated fetal bovine serum, 25 mM KCl, 2 mM glutamine, and 100 μg/mL gentamicin, and then placed on 35-mm glass-bottom dishes (MatTek, Ashland, MA, USA) pre-coated with poly-L-lysine, at a density of ~10^6^ cells/mL (2 mL of cell suspension/dish). After incubation for 3 h, the medium was changed to Neurobasal™-A medium containing B-27 supplement, 2 mM GlutaMAX™-I and 100 μg/mL gentamicin.

In the second experiment, for the qPCR analysis, primary cultures of rat cortical neurons were prepared as described above from the brains of the *Wfs1* KO rats (Sprague-Dawley IGS rats, Charles River, Wilmington, MA, USA) and CD® (Sprague-Dawley) IGS rats (Charles River) as controls (not wild-type littermates). After plating (approximately 3.5 × 10^6^ cells on 60-mm dish), the cells were incubated for 24 h (1 day) or 48 h (2 days) and washed with PBS and resuspended in Trizol, after which the cells were immediately frozen in liquid nitrogen. The samples were kept at −80 °C until RNA isolation.

### 2.5. Transfection with Wfs1 SiRNA

On the third day after plating, the rat primary cortical neurons (plated at lower density, 2.5 × 10^5^ cells/mL) were transfected with 20 nM validated siRNA against *Wfs1* (Sigma-Aldrich: SASI_Rn02_00265296 Rat NM_031823) using the N-TER™ Nanoparticle siRNA Transfection System (Sigma-Aldrich, St. Louis, MO, USA).

Briefly, a mixture of target or scrambled siRNA (20 nM) diluted in siRNA buffer and N-TER transfection reagent diluted in ddH_2_O was preincubated at RT for 20 min. The growth medium was then replaced with Opti-MEM I containing the target or scrambled siRNA mixture. After a 3 h incubation at 37 °C, the Opti-MEM I was changed to Neurobasal™-A medium containing B-27 supplement, 2 mM GlutaMAX™-I, and 100 μg/mL gentamicin. After transfection, the cells were incubated for 48 h at 37 °C in a humidified 5% CO2/95% air incubator before cell lysis for RNA isolation.

### 2.6. RNA Isolation, cDNA Synthesis and Gene Expression Analyses

The lower lung lobe and left ventricle of the heart were homogenized using a Precellys lysing Kit CK14 (Bertin Instruments, Montigny-le-Bretonneux, France) and a Precellys homogenizer (Bertin Instruments). The total RNA from the tissue lysates was isolated using Direct-zol RNA MiniPrep (Zymo Research, Irvine, CA, USA) and from the rat primary cortical neurons using the Qiagen RNeasy Mini Kit, according to the manufacturers’ protocol. The RNA was reverse-transcribed using random hexamers and SuperScript™ III Reverse Transcriptase (Invitrogen, Waltham, MA, USA).

The qPCR was performed on a QuantStudio 12K Flex Real-Time PCR System (Applied Biosystem, Waltham, MA, USA) using the Taqman Gene Expression Mastermix (Thermo Fisher Scientific, Waltham, MA, USA) and the following TaqMan Gene Expression Assays: *Ace* (Rn00561094_m1), *Ace2* (Rn01416293_m1), *Agtr1a* (Rn02758772_s1), *Agtr1b* (Rn02132799_s1), *Agtr2* (Rn00560677_s1), *Bdkrb1* (Rn02064589_s1), *Bdkrb2* (Rn01430057_m1), *Mas1* (Rn00562673_s1) and *Wfs1* (Rn00582735_m1). In all the gene expression experiments, the amount of the target gene was normalized to *Hprt1* (Hypoxanthine-guanine phosphoribosyltransferase; Rn01527840_m1) or *Tbp* (TATA box binding protein, Rn01455648_m1) as an endogenous reference control by means of the 2^−ΔCt^ method [50].

### 2.7. Peptide Level Determination from the Blood Serum

The serum peptide levels were measured using specific ELISA kits for angiotensin-(1–7) (#CSB-E14241r, Cusabio, Houston, TX, USA), angiotensin-(1–9) (#BM-EKU08764, Hölzel Biotech, Köln, Germany), angiotensin II (#ADI-900-204, Enzo Life Sciences), aldosterone (#ADI-900-173, Enzo Life Sciences, Farmingdale, NY, USA), bradykinin (#ADI-900-206, Enzo Life Sciences) and renin 1 (#RAB1162, Sigma-Aldrich), according to the manufacturer’s instructions.

### 2.8. Na^+^ and K^+^ Level Detection from the Blood Serum

To detect possible electrolyte disturbances induced by Wfs1 deficit or changes due to LIR, VPA or their co-treatment, the serum concentration of the physiologically important ions, sodium (Na^+^) and potassium (K^+^), was measured. For the quantification of Na^+^ and K^+^ from the blood serum, an Ion-Selective Electrode (ISE) indirect Na-K-Cl for Gen.2 package insert of the Roche/Hitachi cobas c (Roche Diagnostics, Basel, Switzerland) on a Cobas C501 analyzer was used, based on the manufacturer’s instructions.

### 2.9. Statistical Analysis

GraphPad Prism version 5 software (GraphPad Software Inc., San Diego, CA, USA) and the STATISTICA 8 package (StatSoft Inc, Tulsa, OK, USA) were used for the statistical analysis. The data were compared using factorial ANOVA followed by Fisher’s LSD post hoc tests or the unpaired *t* test. The data are presented as the mean and standard error of the mean (±SEM); a *p* value < 0.05 was considered statistically significant.

## 3. Results

### 3.1. Agtr2, Agtr1b and Bdkrb1 Are Downregulated in a Rat Model of Wolfram Syndrome

To observe the changes in the expression of RAAS, the key components of this system were analyzed from the lower lung lobe and left ventricle of the heart using qPCR.

**Figure 1:**
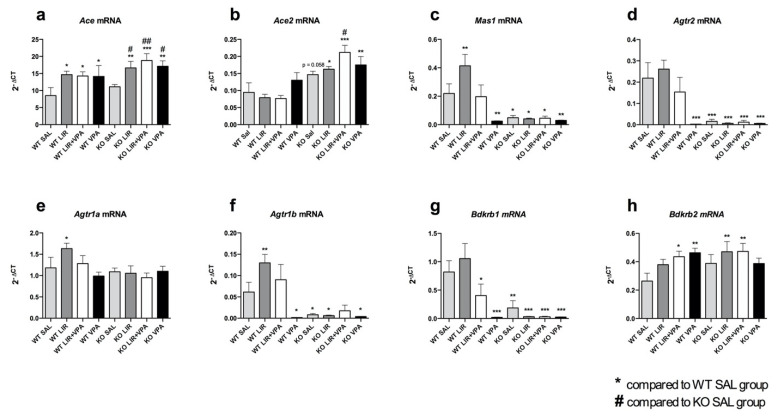
In the lungs, in *Wfs1*-deficiency there is tremendous downregulation of *Mas1*, *Agtr2*, *Agtr1b* and *Bdkrb1*. Gene expression analyses were performed on the lungs of 3.5–4-month-old animals after 8 days of treatment with liraglutide (LIR), valproate (VPA), liraglutide + valproate (LIR + VPA), or saline (SAL). The relative expression level was detected for (**a**) *Ace* mRNA, (**b**) *Ace2* mRNA, (**c**) *Mas1* mRNA, (**d**) *Agtr2* mRNA, (**e**) *Agtr1a* mRNA, (**f**) *Agtr1b* mRNA, (**g**) *Bdrkb1* mRNA and (**h**) *Bdrkb2* mRNA. Gene expression level is presented as 2^−ΔCT^ relative to the housekeeper *Tbp*. Data were compared using factorial ANOVA, followed by Fisher’s LSD post-hoc tests; * *p* < 0.05; ** *p* < 0.01; *** *p* < 0.001 compared to WT vehicle (SAL) group and # *p* < 0.05; ## *p* < 0.01 compared to KO vehicle (SAL) group. The data are presented as the mean ± SEM, *n* = 7–8 per group.

**Figure 2:**
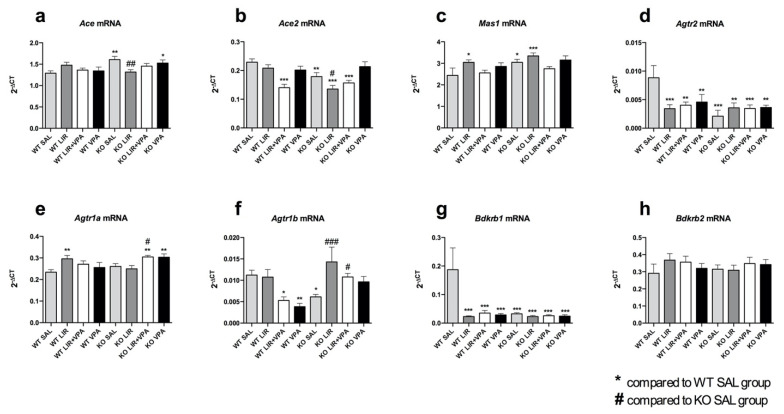
In the heart, in *Wfs1*-deficiency there is extensive downregulation of *Agtr2* and *Bdkrb1*. Gene expression analyses were performed from the heart of 3.5–4-month-old animals after 8 days of treatment with liraglutide (LIR), valproate (VPA), liraglutide + valproate (LIR + VPA), or saline (SAL). The relative expression level was detected for (**a**) *Ace* mRNA, (**b**) *Ace2* mRNA, (**c**) *Mas1* mRNA, (**d**) *Agtr2* mRNA, (**e**) *Agtr1a* mRNA, (**f**) *Agtr1b* mRNA, (**g**) *Bdrkb1* mRNA and (**h**) *Bdrkb2* mRNA. Gene expression level is presented as 2^−ΔCT^ relative to the housekeeper *Tbp*. Data were compared using factorial ANOVA followed by Fisher’s LSD *post-hoc* tests; * *p* < 0.05; ** *p* < 0.01; *** *p* < 0.001 compared to WT vehicle (SAL) group and # *p* < 0.05; ## *p* < 0.01; ### *p* < 0.001 compared to KO vehicle (SAL) group. The data are presented as the mean ± SEM, *n* = 8 per group.

It was found that *Agtr2*, *Agtr1b* and *Bdkrb1* mRNA expression levels, both in the lungs (*p*_Agtr2_ < 0.001, *p*_Agtr1b_ < 0.05, *p*_Bdkrb1_ < 0.01) and hearts (*p*_Agtr2_ < 0.001, *p*_Agtr1b_ < 0.05, *p*_Bdkrb1_ < 0.001) of the WS rats, were substantially lower compared to their WT littermates (Figure 1d,f,g and Figure 2d,f,g). Similarly, the expression of *Mas1* was drastically downregulated in the WS animals, but in the lungs only (*p* < 0.05) (Figure 1c); in the heart, by contrast, *Mas1* was slightly upregulated (*p* < 0.05) (Figure 2c). Conversely, *Ace* expression was increased in the hearts (*p* < 0.01) (Figure 2a) as well as being slightly elevated in the lungs of the WS animals (Figure 1a), albeit insignificantly. In addition, *Ace2* expression also tended to be increased in the lungs (*p* = 0.058) (Figure 1b), but not in the hearts, of the WS animals, while their *Ace2* expression was decreased compared to the WT rats (*p* < 0.01) (Figure 2b). There were no significant changes in the expression levels of *Agtr1a* and *Bdkrb2* between the two genotypes (Figure 1e,h and Figure 2e,h). These genotype-dependent changes in gene expression were more pronounced in the lung, where *Wfs1* expression was shown to be higher compared to the heart.

In summary, significant differences were revealed in the basal level of these genes between the wild-type and *Wfs1* KO rats. Moreover, there were considerable discrepancies between the lungs and the heart tissues.

### 3.2. Agtr2 and Bdkrb1 Expression Is Downregulated in Primary Cortical Neurons of WS Rat

To check the relevance of the difference observed in the expression of *Agtr2* and *Bdkrb1* in the lungs and heart, the expression of *Agtr2*, *Bdkrb1* and *Wfs1* were analyzed in the rat primary cortical neurons of the WS rats.

**Figure 3:**
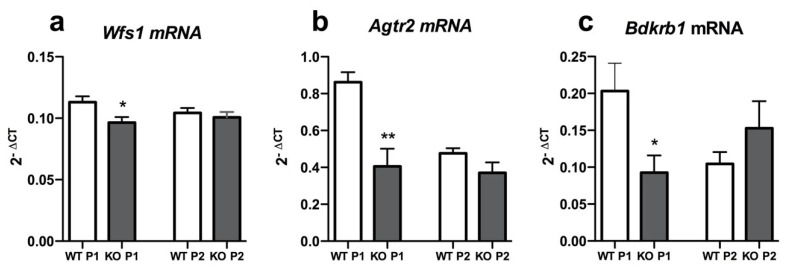
The expression of *Agtr2* and *Bdkrb1* is downregulated in in vitro primary cortical neurons from *Wfs1* KO rats. Rat primary cortical neurons were prepared using the brains of wild-type control or *Wfs1* KO rats and cells were cultured 24 h (P1) or 48 h (P2) post-culturing. Gene expression analysis was performed to measure the relative expression level of (**a**) *Wfs1* mRNA, (**b**) *Agtr2* mRNA and (**c**) *Bdkrb1* mRNA. Gene expression level is presented as 2^−ΔCT^ relative to the housekeeper *Hprt*. Data were compared using unpaired *t* test; * *p* < 0.05, ** *p* < 0.01 compared to respective WT group. The data are presented as the mean ± SEM, *n* = 6–7 per group.

We observed that in the one-day-old primary cortical cells from the WS rats, the expression of *Agtr2* and *Bdkrb1* was again significantly lower compared to the cells from the control rats (*p* < 0.01 and *p* < 0.05, respectively) (Figure 3). At the same time, we observed that *Wfs1* was slightly downregulated in the 24 h WS cortical neurons compared to the control group (*p* < 0.05). In the two-day-old primary cortical neurons from the control rats, the expression of the receptors was decreased to the level of those seen in the WS rats. It may be speculated that the expression of *Agtr2* and *Bdkrb1* in WT cells is increased in response to the stress accompanying the dissection of cells and seeding them into the cell culture, but not in Wfs1 KO cells.

### 3.3. Agtr2 and Bdkrb1 Expression Is Downregulated in Wfs1 Knock-Down

In order to establish whether *Wfs1* knock-down affects the expression of *Agtr2*, *Bdkrb1*, *Ace* and *Ace2* in vitro, the rat primary cortical neurons were transfected with *Wfs1* siRNA using the N-TER Nanoparticle siRNA transfection system. In addition, when culturing the cortical neurons with the method used for the qPCR experiments, it was observed that there were significantly fewer KO cells in the P1 culture compared to WT cells, indicating that some of the KO cell populations died. Thus, using WT cells to knock down *Wfs1* using siRNA, the differences in cell populations should be also minimal.

**Figure 4:**
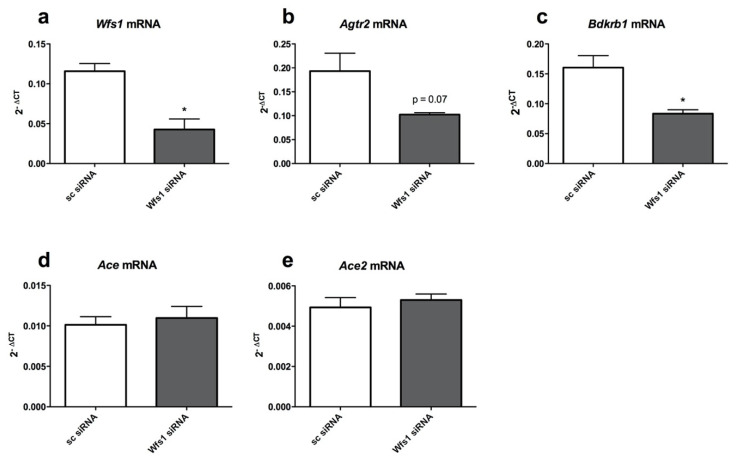
The expression of *Agtr2* and *Bdkrb1* is downregulated in in vitro *Wfs1* knock–down. Rat primary cortical neurons were transfected with control or *Wfs1* siRNA using the N-TER nanoparticle siRNA transfection system and gene expression analysis was performed to measure the relative expression level of (**a**) *Wfs1* mRNA, (**b**) *Agtr2* mRNA, (**c**) *Bdkrb1* mRNA, (**d**) *Ace* mRNA and (**e**) *Ace2* mRNA. Gene expression level is presented as 2^−ΔCT^ relative to the housekeeper *Hprt*. Data were compared using unpaired *t* test; * *p* < 0.05 compared to scramble siRNA. The data are presented as the mean ± SEM, *n* = 3 per group.

The measurement of the relative expression levels of *Wfs1*, *Agtr2*, *Bdkrb1*, *Ace* and *Ace2* were performed using the primary cortical neurons. The expression of *Wfs1* in the neurons was reduced by more than a half after transfection with *Wfs1* siRNA (*p* < 0.05) (Figure 4a). Moreover, similarly to *Wfs1*, the expression of both *Agtr2* (*p* = 0.07) and *Bdkrb1* (*p* < 0.05) was also decreased in the *Wfs1*-deficient neurons approximately by a half (Figure 4b,c), suggesting that the expression of these receptors might be dependent on the level of *Wfs1*. The expression of *Ace* and *Ace2* displayed no change in the *Wfs1* knock–down (Figure 4d,e). Treatment with *Wfs1* siRNA displayed no effect on the expression of *Hprt*, the housekeeper used in the expression analysis. Secondly, although the primary culture of cortical neurons may contained some glial cells, both types of cells expressed RAAS and KKS components.

These results show that, in vivo in the lungs and in the hearts of the *Wfs1*-deficient rats and in vitro in the primary cortical neurons from the brains of the WS rats, the expression of *Agtr2* and *Bdkrb1* was also downregulated in vitro in the Wfs1 knock-down of the rat primary cortical neurons.

### 3.4. Liraglutide and VPA Have a Profound Effect on the Expression of RAAS Components

To explore how acute LIR, VPA or their co-treatment modulate the function of the RAAS and KKS, gene expression analyses from the lower lung lobe and left ventricle of the heart were performed.

In the lungs, regardless of genotype, treatment with VPA drastically downregulated the expression of *Mas1* (*p* < 0.01), *Agtr2* (*p* < 0.001), *Agtr1b* (*p* < 0.05) and *Bdkrb1* (*p* < 0.001) (Figure 1c,d,f,g). However, in the WT animals, the suppressing effect of VPA was reversed by its co-administration with LIR, while this effect of LIR on these genes was absent in *Wfs1* KO (Figure 1c,d,f,g). In the lungs of the WT animals, LIR alone tended to increase all mRNA levels except *Ace2* compared to saline group, even though this difference was statistically significant only in the case of *Ace* (*p* < 0.05), *Mas1* (*p* < 0.01), *Agtr1a* (*p* < 0.05) and *Agtr1b* (*p* < 0.01) (Figure 1a,c,e,f). In the WS rats, this expression-elevating effect of LIR was absent, except for in *Ace* (*p* < 0.05) and *Bdkrb2* (*p* = 0.26) (Figure 1a,h).

In the hearts of the WT animals, all the treatments downregulated the expression of *Agtr2* (*p* < 0.001 and *p* < 0.01, LIR and LIR + VPA/VPA, respectively) and *Bdkrb1* (*p* < 0.001, all treatments), whereas *Agtr1b* was downregulated by VPA (*p* < 0.01) and LIR + VPA co-treatment (*p* < 0.05). Moreover, unlike in the observation of the lungs, LIR + VPA co-treatment did not reverse the VPA treatment-induced decline in *Agtr2*, *Agtr1b* and *Bdkrb1* expression in the hearts of the WT animals (Figure 2d,f,g). Additionally, *Agtr1b* expression levels in the heart were elevated in response to all the treatments in the *Wfs1* KO (*p* < 0.001 and *p* < 0.05, LIR and LIR + VPA, respectively; *p* = 0.12 for VPA) but not in the WT animals (Figure 2f). As seen in the lungs, LIR displayed a modest increasing effect on the expression of *Ace* (*p* = 0.053), *Mas1* (*p* < 0.05), *Agtr1a* (*p* < 0.01) and *Bdkrb2* (*p* = 0.13) (Figure 2a,c,e,h) and no effect on *Ace2* (Figure 2b) in the hearts of the WT animals. In response to acute LIR or LIR + VPA co-treatment, the expression levels of *Ace* and *Ace2* were slightly increased in the lungs (*p*_Ace_ < 0.05, *p*_Ace2_ = and *p_Ace_* 0.001, *p_Ace2_* = 0.05, LIR and LIR + VPA, respectively) and, by contrast, slightly decreased in the heart (*p_Ace_* < 0.01, *p_Ace2_* < 0.05 for LIR and no statistical significance for LIR + VPA) of *Wfs1* KO. The treatment demonstrated no remarkable effect on *Agtr1a* (Figure 1e and Figure 2e) and *Bdkrb2* (Figure 1h and Figure 2h) mRNA levels, neither in the lungs nor in the hearts of the *Wfs1* KO animals.

Overall, it can be concluded that acute pharmacological challenge with LIR and VPA has a notable modulating impact on the expression of RAAS and KKS components and this is affected by genotype as well as the tissue type involved.

### 3.5. The Basal Level of Bradykinin Is Increased and Aldosterone Decreased in Wfs1 KO Rats

**Figure 5:**
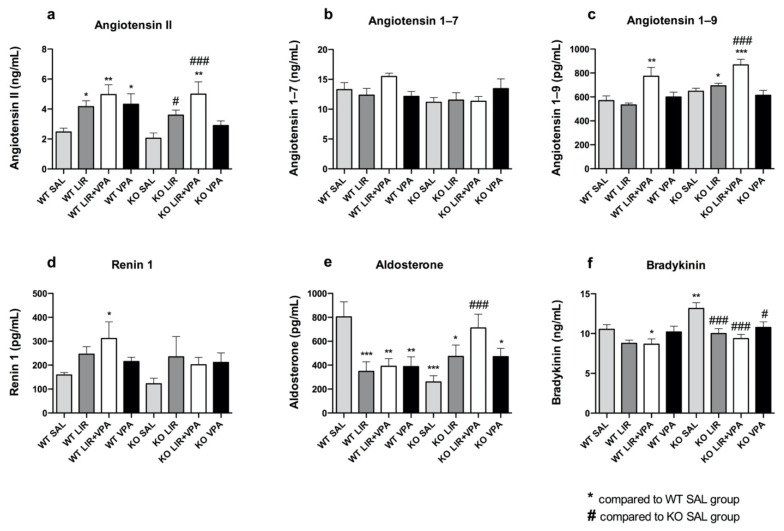
*Wfs1* deficiency leads to drastic decrease in aldosterone and increase in bradykinin serum levels. Neurohormone levels were measured from the blood serum of 3.5–4-month-old animals after 8 days of treatment with liraglutide (LIR), valproate (VPA), liraglutide + valproate (LIR + VPA), or saline (SAL). ELISA was used to measure the serum level of (**a**) angiotensin II, (**b**) angiotensin 1–7, (**c**) angiotensin 1–9, (**d**) renin 1, (**e**) aldosterone and (**f**) bradykinin. Data were compared using factorial ANOVA, followed by Fisher’s LSD tests; * *p* < 0.05; ** *p* < 0.01; *** *p* < 0.001 compared to WT vehicle (SAL) group and # *p* < 0.05; ### *p* < 0.001 compared to KO vehicle (SAL) group. The data are presented as the mean ± SEM, *n* = 7–8 per group.

It was further tested whether genotype and acute LIR or VPA treatment impact on the levels of aldosterone and bradykinin, the main executors of RAAS and KKS. Therefore, the serum peptide levels were analyzed. It was revealed that the level of aldosterone, the downstream facilitator of RAAS, was nearly four times lower in the *Wfs1* KO animals compared to the WT (*p* < 0.001). Treatment with LIR and VPA tended to increase aldosterone levels and LIR + VPA co-treatment normalized the level seen in *Wfs1* KO, increasing it by approximately three times (*p* < 0.001) (Figure 5e). By contrast, bradykinin was increased in the serum of the *Wfs1* KO (*p* < 0.01); however, both LIR (*p* < 0.001) and VPA (*p* < 0.05) and their combination (*p* < 0.001) decreased bradykinin to the basal level in the WT animals (Figure 5f). This is in concordance with the treatment-dependent decrease in *Bdkrb1* expression levels (Figure 1g and Figure 2g) [51]. Additionally, the level of angiotensin II was increased by LIR, VPA or LIR + VPA, both in the *Wfs1* KO (*p*_LIR_ < 0.05, *p*_LIR + VPA_ < 0.001, *p*_VPA_ = 0.25) and in the WT (*p*_LIR_ < 0.05, *p*_LIR + VPA_ < 0.01, *p*_VPA_ < 0.05) (Figure 5a), as well as the level of renin (Figure 5d, statistically insignificant), which forms angiotensin I, the precursor of angiotensin II. Further, LIR + VPA co-treatment elevated the serum levels of Ang-(1–9) independent of genotype (*p* < 0.01 and *p* < 0.001, in the WT and *Wfs1* KO, respectively) (Figure 5c). In the case of Ang-(1–7) a slight, albeit statistically insignificant, increase was observed in response to the co-treatment in the WT, but not in the WS animals (Figure 4b). 

Altogether, aldosterone levels were decreased and bradykinin levels increased in the rat model of WS compared to the control group. The LIR or LIR + VPA acute treatment-induced increase in serum angiotensin II and aldosterone levels and decrease in bradykinin concentrations indicate that all these treatments enhance ACE activity. Serum Ang-(1–9) levels were increased in both the genotypes treated with the combination of LIR and VPA, suggesting more active ACE2.

### 3.6. Genotype and Drug Treatment Had no Effect on Na^+^ and K^+^ Levels

The RAAS is adjusted in response to changes in blood pressure and levels of Na^+^ and K^+^. The RAAS is activated when there is low Na^+^ and/or high K^+^ [52,53]. Disturbances in the RAAS system may reflect changes in electrolyte levels, or vice versa. Thus, to evaluate how the major RAAS-involved ions are affected by the WS genotype and in response to acute treatment with LIR and/or VPA, we measured the blood serum levels of Na^+^ and K^+^. 

**Figure 6:**
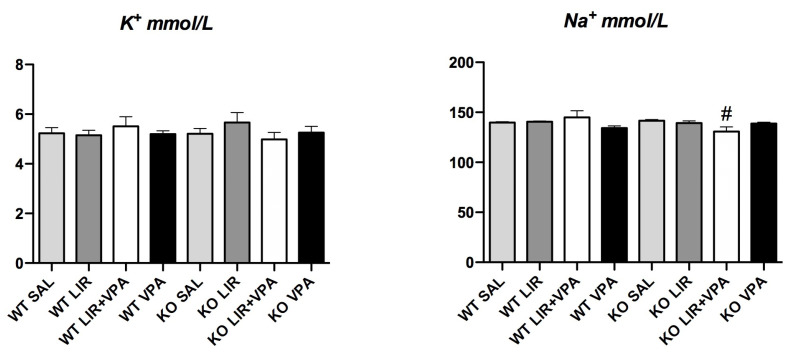
Na^+^ and K^+^ levels measured in 3.5–4-month-old animals’ blood serum after 8 days of treatment with liraglutide (LIR), valproate (VPA), liraglutide + valproate (LIR + VPA), or saline (SAL). Electrolyte levels were determined using Ion-Selective Electrode (ISE) indirect Na-K-Cl for Gen.2 package insert of the Roche/Hitachi cobas c systems (Roche Diagnostics) and are presented as mmol/L. Data were compared using factorial ANOVA followed by Fisher’s LSD post hoc tests; # *p* < 0.05 compared to KO vehicle (SAL) group. The data are presented as the mean ± SEM, *n* = 6–8 per group.

To our surprise, there were no differences detected in the levels of Na^+^ or K^+^ between the WT and WS rats and neither LIR or VPA treatment nor their combination demonstrated a considerable effect on the levels of these electrolytes (Figure 6).

## 4. Discussion

To date, there is no clear understanding of the exact mechanism of the development of Wolfram syndrome (WS). Therefore, every new piece in this puzzle is of utmost importance.

The anti-diabetic drug GLP-1R agonist liraglutide (LIR) and the anti-epileptic drug valproate (VPA) have shown promising results in preclinical studies and are assessed in upcoming clinical trials against WS progression (https://thesnowfoundation.org/clinical-trials/, accessed on 7 April 2021, ClinicalTrials.gov Identifier: NCT03717909 [54]). However, it is not known through which targets LIR and/or VPA exert their inhibitory effect on the progression of WS. Both of those drugs have been shown to affect the expression of RAAS components [17,18]. WFS1 and its physiological role have been thoroughly studied, yet the role of WFS1 in RAAS and bradykinin pathways (kallikrein-kinin system) has never been evaluated.

Thus, in the current study, we used 3.5–4 month-old male *Wfs1* KO rats and their WT littermates to detect early changes in RAAS and KKS before the animals could fully develop the WS phenotype [3]. The animals received a daily dose of LIR, VPA, LIR + VPA or SAL for eight consecutive days. We analyzed the gene expression of different RAAS and KKS components from the lungs and heart, where high levels of WFS1 expression have been described [41,42,43], and from the rats’ primary cortical neurons. In addition, we measured the serum levels of the main hormones involved in these systems. Here, we show that RAAS and KKS are dysregulated in *Wfs1*-deficiency and that LIR and VPA have a substantial modulating effect on these systems.

We found that the expression of key receptors of RAAS, namely *Agtr2* and *Bdkrb1*, were drastically downregulated in *Wfs1* deficiency, while neither treatment was able to affect these expression levels in the WS rats. Similar reductions were seen in the expression of *Agtr1b* and *Mas1,* the latter only in the lungs; again, acute treatment with LIR or VPA had no effect on their expression in the lungs. However, in the lungs of the WT animals, treatment with VPA clearly downregulated the expression of *Agtr2*, *Mas1*, *Agtr1b* and *Bdkrb1*; all these effects were reversed by the co-administration of LIR. In the hearts of the WT animals, all the treatments downregulated *Agtr2* and *Bdkrb1* gene expression, whereas *Agtr1b* was downregulated by VPA only. Again, this effect was pharmacologically antagonized by LIR. These findings coincide with the results of a previous study, which demonstrated that VPA can downregulate *Agtr2* gene expression [55].

Furthermore, the WFS1-dependent expression of *Agtr2* and *Bdkrb1* was confirmed in vitro in rat primary cortical neurons. The expression of *Agtr2* and *Bdkrb1* was downregulated in one-day-old cortical neurons from WS rats, as well as after *Wfs1* knock-down in cortical neurons by siRNA. Although in the two-day-old cortical neurons from the WT the expression levels decreased to the levels seen in the WS, this might be attributed to the stressful conditions. Under normal conditions, the expression of *Wfs1* is constant and is degraded by ubiquitin ligase SMURF1 if it is not needed. In stressful situations, SMURF1 is degraded and WFS1 can perform its function [56]. WFS1 is a negative regulator of ER stress and it may be speculated that functional WFS1 induces *Agtr2* and *Bdkrb1* gene expression, thereby helping to cope with stressful conditions. Collectively, we can conclude from our in vivo and in vitro gene expression data that in the rat model, knock-down or loss of functional WFS1 seems to lead to the downregulation of *Agtr2* and *Bdkrb1* expression. However, neither LIR nor VPA restored the normal expression of these genes.

Therefore, we also analyzed the serum peptide levels, including aldosterone and bradykinin (the active peptide of KKS). We observed that they were significantly altered in the WS rats. Aldosterone is known to have pro-fibrotic and pro-inflammatory effects that, among others, are mediated by inflammation and oxidative stress [57]. Low plasma aldosterone levels have been described in patients with type 1 diabetes [58] and elevated aldosterone is associated with type 2 diabetes by affecting insulin sensitivity [59,60]. Thus, surprisingly, the aldosterone levels were nearly four times lower in the WS compared to the WT rats. Although the aldosterone levels were substantially lower in the WS rats, this was not reflected in the blood serum electrolyte content, as we had expected. Contrary to the aldosterone results, we detected an elevation in bradykinin levels in the WS rats compared to the WT.

Ang I is processed by ACE into Ang II [28] and Ang II stimulates the release of aldosterone from the adrenal glands via the activation of AGTR1 and AGTR2 receptors [61,62]. Acute treatment with LIR, VPA and especially LIR + VPA increased the Ang II and normalized the aldosterone and bradykinin concentrations in the blood serum of the WS rats. Keeping in mind that bradykinin is mostly degraded by ACE [38,39], we can conclude that all these treatments enhanced ACE activity in the *Wfs1* KO rats. It can be hypothesized that the downregulation of bradykinin might contribute to the reduction of neuroinflammation and ER stress, as was observed in the WS rats’ response to LIR [11]. In the WT rats, all the treatments decreased aldosterone levels. Similar results were reported using LIR and exenatide in healthy subjects, where the acute administration of LIR tended to suppress and chronic dosing to increase aldosterone, while a single therapeutic dose of exenatide led to the clear suppression of aldosterone [63,64].

Previous studies have shown that LIR or other GLP-1R agonists increase ACE2 activity [17]. ACE2 processes Ang I to Ang-(1–9); therefore, Ang-(1–9) levels reflect ACE2 activity. Our results indicate that LIR and VPA alone do not elevate serum Ang-(1–9) levels; their combination, on the other hand, increased Ang-(1–9) levels independent of genotype, implying increased ACE2 activity. All this suggests that VPA and LIR co-treatment increases ACE and ACE2 activity more than administration of either of the drugs separately. Hence, we can conclude that co-treatment has a stronger impact on the RAAS system.

Here, we demonstrated tremendous downregulation of *Bdkrb1* and *Agtr2*, both of which are known to affect bradykinin sensitivity. Bradykinin is involved in inflammatory processes [65] and has been shown to induce BDKRB1-mediated Ca^2+^-dependent microglial migration in the CNS that relies on the reverse mode of sodium-calcium exchanger (NCX1) [36]. In the cardiomyocytes of WS rats, NCX1 is substantially downregulated [66]. Furthermore, after bradykinin stimulation, fibroblasts from WS patients release lower amounts of Ca^2+^ from the ER compared to control samples [67], indicating the loss of bradykinin receptor sensitivity and NCX1 activity. Beside the role in RAAS, AGTR2 promotes axonal regeneration of the optic nerve in adult rats [32] and AGTR2 hetero-dimerization with TRKB and BDKRB2, enhancing receptor-mediated signal transduction, has also been described [68,69]. Since AGTR2 has been shown to form active heterodimers with BDKRB2 [69] and we observed a clear decrease in *Agtr2* expression in *Wfs1*-deficiency, it can be speculated that despite the normal *Bdkrb2* expression in the WS rats its sensitivity might have been reduced. Altogether, *Bdkrb1* downregulation and a speculative decrease in BDKRB2 receptor sensitivity may lead to the dysregulation of Ca^2+^ homeostasis and interfere with the oxidative and ER stress and immune response in WS.

## 5. Conclusions

Our results indicate considerable abnormalities in the RAAS and bradykinin systems already at an early stage in a rat model of WS when the primary symptoms have not yet fully developed. RAAS has never been studied in Wolfram syndrome, although, it has been shown that due to severe hydronephrosis and to neurodegeneration, WS patients may have a serious imbalance in natriuretic peptides, which may lead to alterations in RAAS and result in mineralocorticoid deficiency [70,71]. We show for the first time that in *Wfs1*-deficiency, the expression of *Agtr2* and *Bdkrb1* receptors is downregulated both in vitro and in vivo.

Acute treatment with LIR, VPA and LIR +VPA does not normalize the gene expression levels seen in WS rats. Nevertheless, it displays a remarkable modulatory effect on RAAS. The dysregulation of RAAS promotes oxidative stress, inflammation, vascular leakage and arterial stiffness, resulting in the pathophysiology of many diseases, including pulmonary fibrosis, cancer, diabetes and neurodegeneration [19,20,21,22].

So far, WFS1 has been implicated in the regulation of cellular Ca^2+^ homeostasis and ER stress response; however, the exact mechanism remains to be resolved. In light of the data presented in this study, it might be speculated that WFS1 might modulate RAAS and KKS and lead to changes in inflammatory response and the immune system, as well as to disturbances in Ca^2+^ homeostasis and oxidative stress and, thereby, accelerate the progression of WS.

Among other benefits, identifying targets to treat WS would help to clarify the nature of this disease and develop more advanced therapies.

## 6. Limitations of the Study

The main organs involved in the RAAS are the kidneys, lungs, cardiovascular system and the brain. In our study, only lungs and heart tissues from the WS animals were used, based on their abundant *Wfs1* expression. Further study is needed to investigate imbalances in the RAAS in tissues other than the lungs and hearts of WS rats. This is even more important given that, in addition to the well-known systemic RAAS, local RAAS displays complex cross-regulated communication in various tissues.

As already mentioned in the discussion of our results, the primary culture of cortical neurons may contain some glial cells, but to a very limited extent. In addition, both types of cells express RAAS and KKS components.

Furthermore, we are aware of the limitations of our study, as gene expression levels do not always reflect protein levels. Sometimes, protein levels do not correlate with gene expression due to the translation regulation. There is preparedness, but the conditions do not require protein production. Additionally, the presence of protein does not mean it is functional. However, the purpose of this study was to describe changes in the level of gene expression. The future plan is to investigate whether the observed changes in expression levels are also visible at the protein level.

Our aim was to observe whether there are changes in the RAAS system already at the early stage in a rat model of Wolfram syndrome, when the diabetic phenotype is not yet fully developed, and whether LIR and VPA produce an acute effect. Of course, it would be interesting to determine if and how these changes in gene expression and peptide levels altered during aging and through the chronic administration of LIR and VPA.

In addition to age, among other aspects, the changes observed in the RAAS components may also have been affected by the animals’ health and the anesthetics used.

## Data Availability

The datasets generated and/or analyzed in the current study are available from the corresponding authors on reasonable request.
